# Lytic promoter activity during herpes simplex virus latency is dependent on genome location

**DOI:** 10.1128/jvi.01258-24

**Published:** 2024-10-21

**Authors:** Navneet Singh, Sherin Zachariah, Aaron T. Phillips, David Tscharke

**Affiliations:** 1 John Curtin School of Medical Research, The Australian National University, Canberra, Australia; The University of Arizona, Tucson, Arizona, USA

**Keywords:** herpes simplex virus, latency, gene expression

## Abstract

Herpes simplex virus 1 (HSV-1) is a significant pathogen that establishes lifelong latent infections with intermittent episodes of resumed disease. In mouse models of HSV infection, sporadic low-level lytic gene expression has been detected during latency in the absence of reactivation events that lead to production of new viruses. This viral activity during latency has been reported using a sensitive Cre-marking model for several lytic gene promoters placed in one location in the HSV-1 genome. Here, we extend these findings in the same model by examining first, the activity of an ectopic lytic gene promoter in several places in the genome and second, whether any promoters might be active in their natural context. We found that *Cre* expression was detected during latency from ectopic and native promoters, but only in locations near the ends of the unique long genome segment. This location is significant because it is in close proximity to the region from which latency-associated transcripts (LATs) are derived. These results show that native HSV-1 lytic gene promoters can produce protein products during latency, but that this activity is only detectable when they are located close to the LAT locus.

**IMPORTANCE:**

HSV is a significant human pathogen and the best studied model of mammalian virus latency. Traditionally, the active (lytic) and inactive (latent) phases of infection were considered to be distinct, but the notion of latency being entirely quiescent is evolving due to the detection of some lytic gene expression during latency. Here, we add to this literature by finding that the activity can be found for native lytic gene promoters as well as for constructs placed ectopically in the HSV genome. However, this activity was only detectable when these promoters were located close by a region known to be transcriptionally active during latency. These data have implications for our understanding of HSV gene regulation during latency and the extent to which transcriptionally active regions are insulated from adjacent parts of the viral genome.

## INTRODUCTION

Herpes simplex virus 1 (HSV-1) infects around 67% of world population and generally causes mild disease in the form of cold sores (1). For most individuals, HSV-1 disease is infrequent and often asymptomatic; however, that is not always the case and the virus can cause severe diseases such as neonatal herpes and herpes keratitis and encephalitis. Initial HSV-1 infection usually begins in the skin or mucosal epithelium, during which the expression of viral genes occurs in a sequential cascade, categorized as immediate–early (IE), early (E), and late (L), ultimately resulting in the production of new virus particles (2, 3). The late genes are further divided into two subclasses as leaky-late (γ_1_) or true-late (γ_2_), where the expression of the latter strictly occurs following DNA replication (4, 5). The virus quickly spreads to the cell bodies of innervating neurons within the sensory or autonomous nervous system, where latency is established, allowing viral persistence. Virus replication may occur in some neurons during productive infection, but replication is not necessary for the establishment of latency (6, 7). During latency, the HSV-1 genome persists in a non-productive state within the latently infected neurons, while retaining the potential for reactivation.

**Fig 1:**
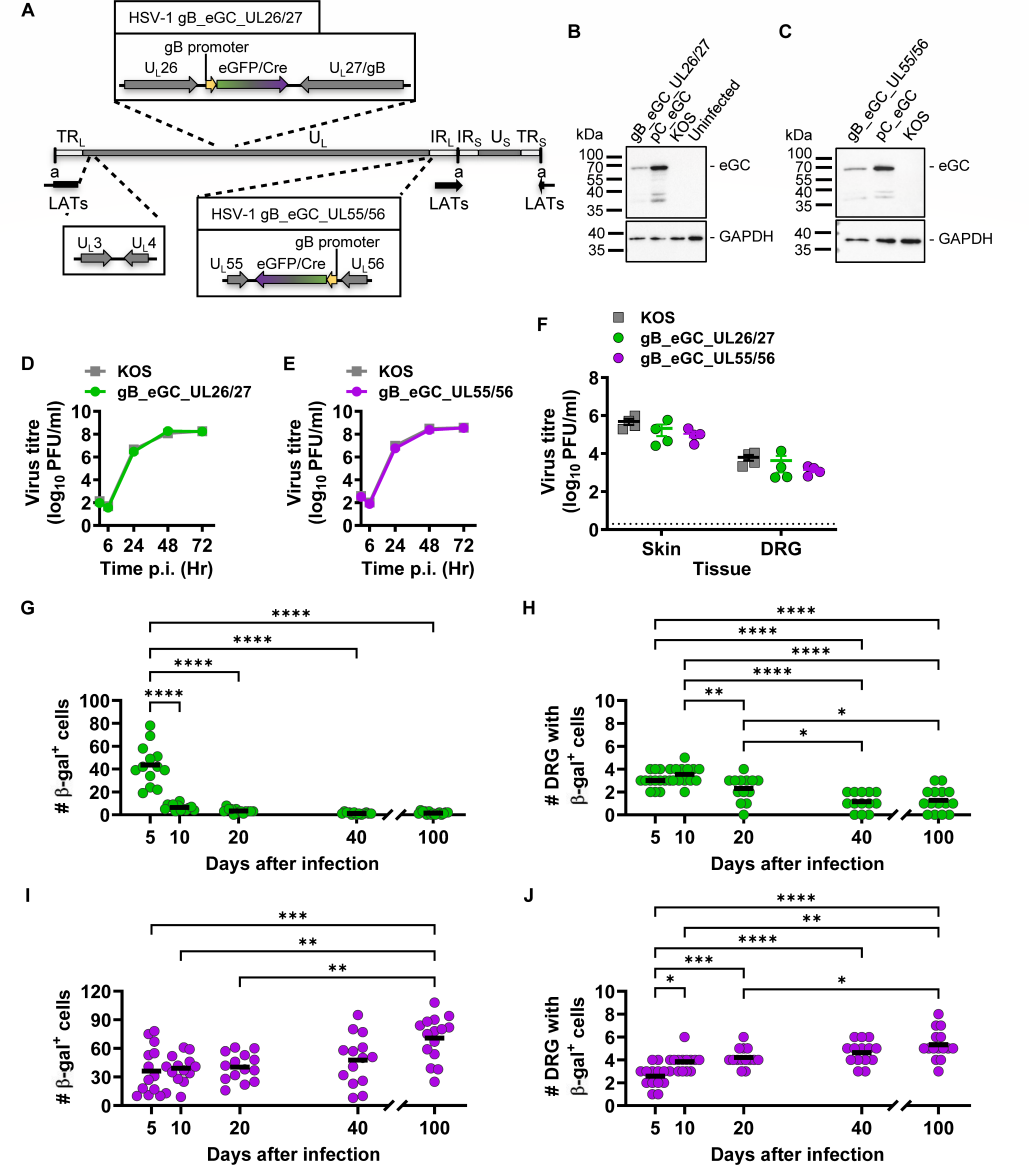
Activity of the ectopic gB promoter from two independent locations. (**A**) Schematic showing the HSV-1 genome (middle; to scale) with unique long (U_L_) and short (U_S_) regions bracketed by repeats (TR_L_/TR_S_; IR_L_/IR_S_), ‘a’ repeats, and position of LATs. The U_L_26/U_L_27 and U_L_55/U_L_56 regions have been expanded to show the gB_eGFP/Cre expression cassette inserted to generate HSV-1 gB_eGC_UL26/27 (top) and gB_eGC_UL55/56 (bottom), respectively. (**B, C**) Vero cells were either left uninfected or infected with HSV-1 gB_eGC_UL26/27, gB_eGC_UL55/56, KOS, pC_eGC at 10 PFU/cell, and Cre and GAPDH proteins detected by Western blotting as shown. The size of protein fragments from the marker is shown on left. (**D, E**) Replication of recombinants assessed in comparison with parent in Vero cells. Data are shown as mean ± SEM from three replicates (error bars are obscured by data points). (**F**) Mice were infected on the flank with recombinants, and parent viruses and viral loads were measured in skin and DRG after 5 days. Each symbol represents one mouse, and the dotted line signifies the limit of detection (2 PFU/sample). Statistical significance was determined using two-way ANOVA with Tukey’s post-test comparison (unmarked, not significant). (**G-J**) Groups of ROSA26R mice were infected with HSV-1 gB_eGC_UL26/27 (**G, H**) or gB_eGC_UL55/56 (**I, J**), and their DRG collected and stained for beta-galactose (β-gal) expression. Data are pooled from two independent experiments for each virus. The number of β-gal^+^ cells per mouse (**G, I**) and the number of DRG with at least one β-gal^+^ cell (**H, J**) are shown. Differences in means were compared using one-way ANOVA with Bonferroni’s *posttest* to calculate pairwise comparisons (*, *P* < 0.05; **, *P* < 0.01; ***, *P* < 0.001; unmarked, not significant).

The HSV-1 genome as it is packaged in the virion has two segments of unique sequence, termed unique long (U_L_) and unique short (U_S_), each of which is bracketed by repeats at each end (R_L_ and R_S_). By convention, the genome is considered to start with the long segment and genes are numbered from left to right in U_L_ and U_S_ (Fig. 1A). To complete this nomenclature, the repeats that are at the ends of the genome are referred to as being terminal (TR_L_ and TR_S_) and those that join the long and short segments are internal (IR_L_ and IR_S_). Once inside a host cell, the genome becomes a circle, and the long and short segments are able to flip with respect to each other, so there is no biological distinction between internal and terminal repeats. These repeated regions are large enough to encode some genes, meaning these genes are present in two copies, with each copy adjacent to different genes from U_L_ or U_S_.

HSV-1 latency at the level of the whole organism is largely quiescent as spontaneous reactivation events are exceptionally rare (8–10). However, this perspective is challenged when the viral activity is examined at the cellular level. High-level gene expression during latency is limited to noncoding RNAs known as latency-associated transcripts (LATs) and certain micro-RNAs that originate from the LAT region. The expression of LATs begins early during infection, which aligns with the simultaneous establishment of latency and productive infection at the cellular level (7) (11). Studies employing careful PCR and *in situ* hybridizzation analysis have revealed that high levels of LATs are found in only 5 to 30% of latently infected neurons at a given time in latency (12–16). Several reports over the years have provided evidence supporting the presence of lytic transcripts in the latently infected trigeminal ganglia of mice, challenging the traditional view of strict viral quiescence in latency (8, 17–25). One notable report showed that lytic genes belonging to at least one of the classes were expressed in almost two-thirds of infected neurons (21), a far higher frequency than that of neurons exhibiting spontaneous reactivation (26). This suggests that lytic gene transcription is a common phenomenon during latency in the absence of overt reactivation and is likely to be biologically relevant as an increase in viral activity was correlated with a progressive response from the host (21).

There is also evidence that some lytic transcripts may generate proteins during latency, which is from three main sources. First, immune infiltrates consisting primarily of virus-specific and activated CD8^+^ T cells have been found in latently infected sensory ganglia (8, 26–31). Second, the use of Cre reporter mice, such as ROSA26R (32), infected with recombinant viruses that express *Cre*- from lytic gene promoters suggests these promoters are active during latency. In the ROSA26R model, any Cre expression leads to the indelible marking of neurons and acts as a record of historic gene expression (33, 34). In these experiments, accumulation of marked neurons was observed during latency, indicating that lytic gene promoters were able to drive *Cre* expression beyond the acute infection. Promoters for infected cell protein (ICP)47 (U_S_12), ICP6 (U_L_39), and glycoprotein B (gB) (U_L_27), but not ICP0, were able to drive Cre expression during latency (35). Thus, the expression of lytic genes during latency could stem from unsuccessful abortive reactivation events by the virus, incomplete repression of the genome, or more speculatively, a latency-associated gene expression program (36). Finally, a report that analyzed ICP0 mutants concluded that this protein must be expressed during latency based on differences in chromatin and heterochromatin at various promoters and transcript levels of a set of HSV genes at 28 days after infection (24). However, these measures were only carried out at day 28, so it remains difficult to separate a role for ICP0 protein expression in the establishment of latency from a role in latency proper.

A significant limitation of the original Cre marking studies was that the promoters were not examined in their natural location (35). More recently, ICP47 promoter activity in latency was examined in its natural location and when placed between U_L_26 and U_L_27 (U_L_26/U_L_27), and no evidence of Cre expression was detected (37). Importantly however, this study recapitulated previous data showing expression from the ICP47 promoter in latency when placed between U_L_3 and U_L_4 (U_L_3/U_L_4), using the original and several newly made viruses (35, 37). This suggests that the promoter and genome location might be important determinants of the level of protein expression that occurs in latency.

Here, we have systematically investigated whether the expression of lytic genes during latency might be influenced by their location in the HSV-1 genome. This was accomplished by making viruses with expression constructs inserted into multiple sites in the genome, some in their natural (native) context and others in ectopic locations. The results provide evidence of gene expression from HSV lytic gene promoters in their natural context throughout latency and that the level of this activity is influenced by location in the HSV-1 genome.

## RESULTS

### Ectopic gB promoter activity in HSV-1 latency depends on the genomic location

Previous studies have demonstrated that the infection of ROSA26R mice with HSV-1 recombinants expressing Cre under the control of the gB promoter from U_L_3/U_L_4 led to promoter activation and marking of neurons during HSV latency (35). To test the effect of genome location on expression from the gB promoter in latency, we chose two new sites: U_L_26/U_L_27, close to its natural location, and between U_L_55 and U_L_56 (U_L_55/U_L_56) (Fig. 1A). U_L_55/U_L_56 is at the far end of the U_L_ segment, close to IR_L_ from which the LATs are transcribed. This is a different immediate genetic context, but similar distance to the LAT enhancer compared with U_L_3/U_L_4 (Table 1). For each site, we used the same expression cassette published previously (35), with the gB promoter driving an *eGFP/Cre* fusion gene (Fig. 1A). These viruses were named gB_eGC_UL26/27 and gB_eGC_UL55/56, respectively.

**TABLE 1:** Detail of viruses and the distance of promoters / start of Orf from the closest LAT enhancer (LAP2)

Virus (all HSV-1)	Cre expression promoter (location)	Parent virus	Distance from the LAT enhancer (IR_L_/TR_L_)	Reference
KOS	Nil	N/A^*a*^	N/A	(38)
pgB_eGC	gB (U_L_3/U_L_4)	HSV-1 KOS	4.2 kb (TR_L_)	(35)
pC_eGC	CMV IE (U_L_3/U_L_4)	HSV-1 KOS	4.2 kb (TR_L_)	(39)
gB_eGC_UL26/27	gB (U_L_26/U_L_27)	HSV-1 KOS	45.3 kb (TR_L_)	This study
gB_eGC_UL55/56	gB (U_L_55/U_L_56)	HSV-1 KOS	2.6 kb (IR_L_)	This study
UL27-T2A-Cre	gB (natural)	HSV-1 KOS	48.2 kb (TR_L_)	This study
gB_eGTC_UL3/4	gB (U_L_3/U_L_4)	HSV-1 pCmC (39)	4.2 kb (TR_L_)	This study
UL3-T2A-Cre	U_L_3 (natural)	HSV-1 pCmC (39)	3.4 kb (TR_L_)	This study
UL56-T2A-Cre	U_L_56 (natural)	HSV-1 KOS	1.9 kb (IR_L_)	This study
ICP47ins_eGC	ICP47 (natural)	HSV-1 KOS	12.6 kb (TR_L_)	(37)
pICP47_eGC_OG	ICP47 (U_L_3/U_L_4)	HSV-1 KOS	4.2 kb (TR_L_)	(35, 37)
pICP47_eGC_OG26/27	ICP47 (U_L_26/U_L_27)	HSV-1 KOS	45.3 kb (TR_L_)	(37)

^*a*^ N/A, not applicable.

The use of U_L_26/U_L_27 as an insertion site has been shown not to compromise replication or pathogenesis (39), but U_L_55/U_L_56 has not been evaluated. Therefore, both new viruses were characterized carefully. We confirmed the insertion of gB_eGC in the correct region for these new viruses by a whole-genome digest (Fig. S2A and B). We then checked eGFP/Cre fusion protein expression in infected Vero cells by Western blotting with unmodified HSV-1 KOS and a previously characterized virus with the same eGFP/Cre cassette (HSV pC_eGC) (35) as negative and positive controls, respectively (Fig. 1B and C). The eGFP/Cre protein was detected as expected, but there were low-molecular weight fragments, not in the KOS-infected cells, that were likely cleaved or degraded products (Fig. 1B and C). Next, we validated that Cre was functional when expressed by the new viruses using a Cre-reporter cell line Vero SUA (40) (Fig. S3A through F). Finally, we showed that replication of these viruses was the same as for the parent HSV-1 KOS (Fig. 1D and E) and in mice infected by our flank tattoo model (Fig. 1F) (39).

After validating these viruses, they were used to explore gB promoter activity in latency. ROSA26R mice were tattoo-infected on the flank with HSV-1 gB_eGC_UL26/27 or gB_eGC_UL55/56, and at 5, 10, 20, 40, and 100 days after infection, their dorsal root ganglia (DRG) were stained for β-gal activity, and the number of stained neurons was counted (Fig. S4A and B). In the case of HSV-1 gB_eGC_UL26/27, we saw a decrease in the number of β-gal^+^ cells from days 5 to 10, which remained stable thereafter (Fig. 1G). However, the number of DRG with β-gal^+^ cells, which represents the spread of the virus, was similar between days 5 and 10, reduced on days 20 and 40, and remained stable thereafter in latency (Fig. 1H). By contrast, in HSV-1 gB_eGC_UL55/56, the β-gal^+^ cell number was similar for all time points up to day 40 and then was increased at day 100, being significantly higher than that on day 20 and earlier (Fig. 1I). The number of DRGs with at least one β-gal^+^ cell was also increased at day 100 compared with earlier days (Fig. 1J). In summary, we found evidence that the gB promoter was active during latency when driving Cre from U_L_55/U_L_56, but not from U_L_26/U_L_27. Together with previously published results, these data suggest that there is nothing special about the U_L_3/U_L_4 locus that allows expression in latency, but that proximity to IR_L_ and therefore the LAT enhancer is likely to be important.

### Protein expression from the gB promoter in its natural context is undetectable during latency

**Fig 2:**
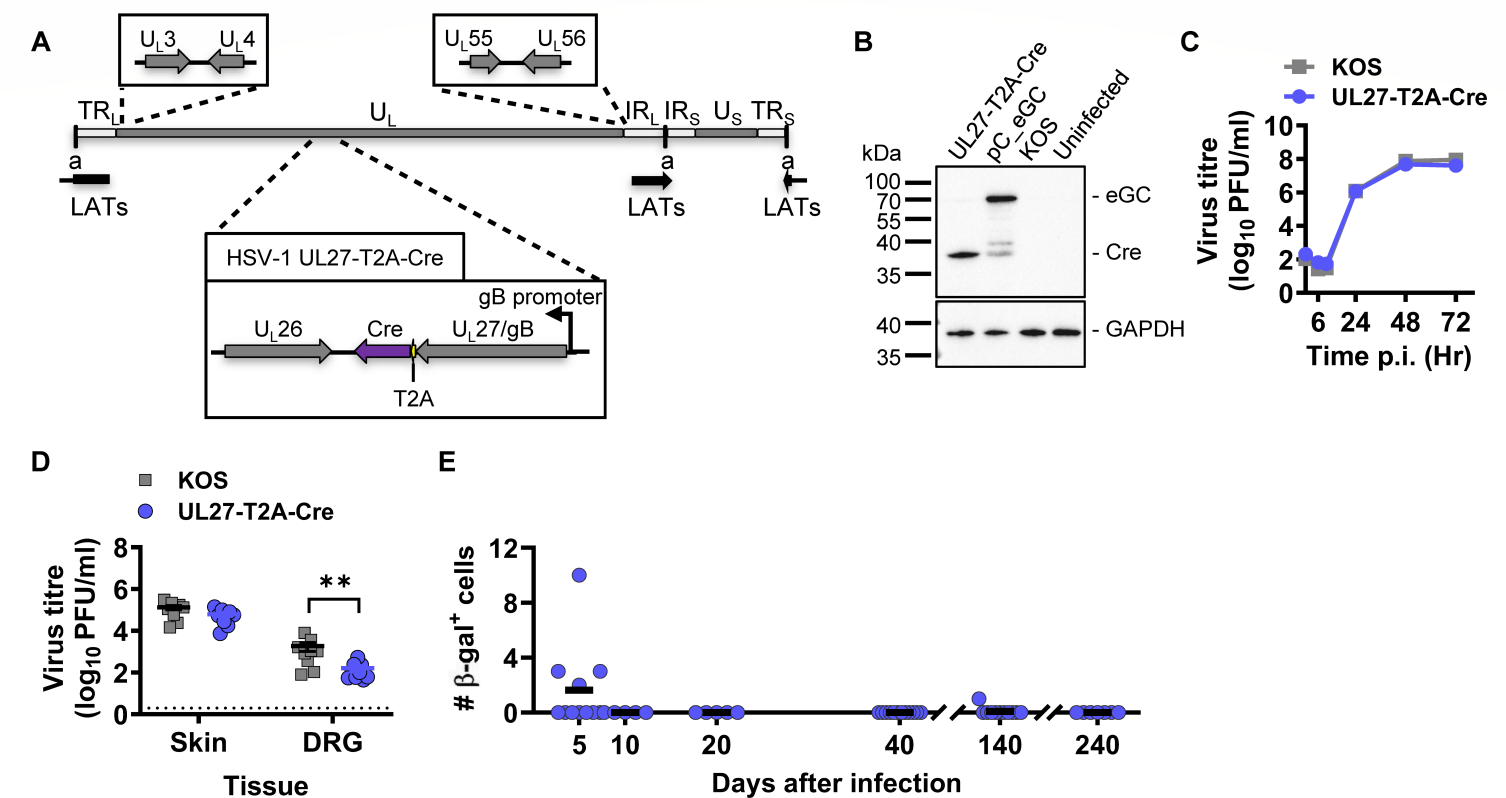
Evaluation of native gB promoter activity. (**A**) Depiction of modifications made to introduce T2A-Cre in the HSV-1 genome (to scale) to make UL27-T2A-Cre. (**B**) Vero cells were either left uninfected or infected with HSV-1 UL27-T2A-Cre, pC_eGC, and KOS at 10 PFU/cell, and Cre and GAPDH proteins were detected by Western blotting. (**C, D**) Replication of recombinant was assessed in comparison with that of the parent *in vitro* (**C**) and *in vivo* (**D**). Symbols and markers are same as in the previous figure. Statistical significance was determined using two-way ANOVA with Tukey’s *posttest* comparison (**, *P* < 0.01; unmarked, not significant). (**E**) Groups of ROSA26R mice were infected with HSV-1 UL27-T2A-Cre, their DRG collected, and stained for β-gal expression. The number of β-gal^+^ cells per mouse is shown, and the data are pooled from two independent experiments. Differences in means were compared using one-way ANOVA with Bonferroni’s *posttest* to calculate pairwise comparisons (unmarked, not significant).

The version of the gB promoter used in experiments thus far was removed from its original context. To enable experiments that maintained the natural position of this promoter, we prepared a virus in which sequences encoding a T2A peptide (41) followed by Cre were added to the 3’ end of the gB open reading frame (called UL27-T2A-Cre) (Fig. 2A). A T2A sequence was chosen because it allows two proteins to be prepared at an equimolar ratio from a single mRNA (42–44).

The expression of gB and Cre as independent proteins was verified by Western blotting, and this virus grew at a similar rate to parent *in vitro* (Fig. 2B and C). Moreover, Cre was functional in Vero SUA reporter cells (Fig. S3). Next, we assessed the growth potential of this virus *in vivo*, in skin and DRGs of C57BL/6 mice, and we found similar viral loads in the skin; however, the titer of the recombinant virus was reduced in the DRGs, compared with the parent virus (Fig. 2D). When ROSA26 mice were infected to determine the native gB promoter activity, we observed a few marked neurons in four out of 11 mice on day 5 and none on days 10, 20, and 40 (Fig. 2E). We decided to extend the experiment to days 140 and 240, instead of day 100 as done previously, and found that the majority of the mice did not have any marked neurons on these days (Fig. 2E). The lack in activity from the native gB promoter in latency is consistent with what was observed for the ectopic promoter placed in the same location, but the marking at day 5 was surprisingly low. This low marking might be due to less virus in neurons, noting the lower-than-expected amounts of virus compared with the parent, but it is possible that the T2A sequence is not behaving as expected *in vivo*.

### Verification of T2A sequences in a recombinant HSV

**Fig 3:**
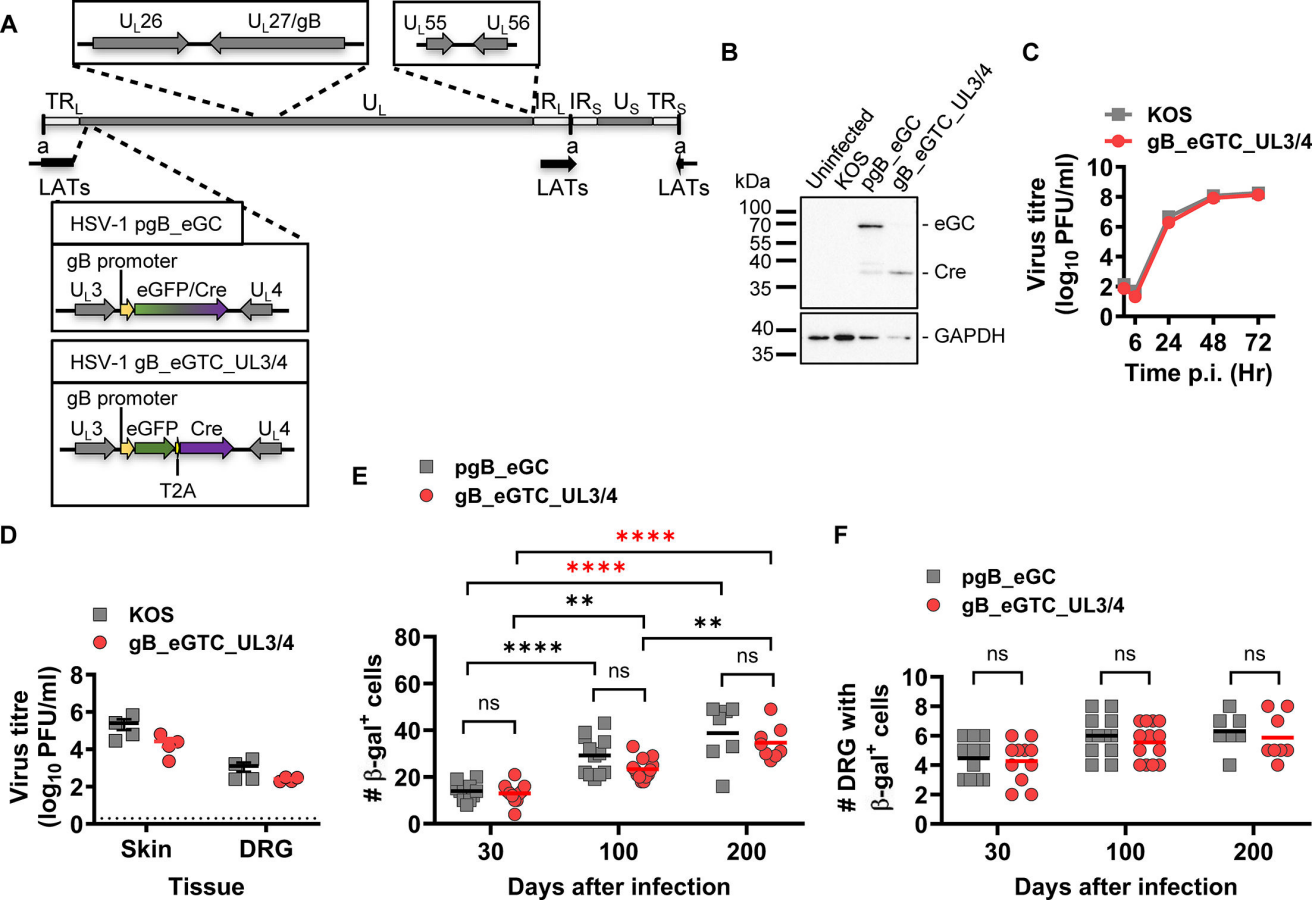
T2A does not affect neuronal marking in the ROSA26R/Cre mouse model. (**A**) Schematic showing modifications made in the HSV-1 genome to generate gB_eGC and gB_eGTC_UL3/4. (**B**) Vero cells were either left uninfected or infected with HSV-1 gB_eGC, gB_eGTC_UL3/4, and KOS at 10 PFUs/cell, and Cre and GAPDH proteins were detected by Western blotting. Controls are the same as in Fig. 1B. (**C, D**) Replication of recombinant was assessed in comparison with that of the parent (same as in Fig. 1D) *in vitro* (**C**) and *in vivo* (**D**). Symbols and markers are same as in the previous figure. Statistical significance was determined using two-way ANOVA with Tukey’s *posttest* comparison (unmarked, not significant). (**E, F**) Groups of ROSA26R mice were infected with HSV-1 gB_eGC or gB_eGTC_UL3/4 at the same time, their DRGs collected, and stained for β-gal expression. The number of β-gal^+^ cells per mouse (**E**) and the number of DRG with at least one β-gal^+^ cell (**F**) are shown. Data are pooled from two independent experiments. Differences in means were compared using two-way ANOVA with Sidak’s *posttest* to calculate pairwise comparisons (**, *P* < 0.01; ****, *P* < 0.0001; ns, not significant; red stars signify differences of a particular note).

To validate the use of T2A sequences in our HSV-1 Cre-marking model, we generated a recombinant virus with a T2A sequence between eGFP and Cre, under the control of the gB promoter inserted in the U_L_3/U_L_4 region (Fig. 3A). This new virus was called gB_eGTC_UL3/4 and was designed to match the previously published HSV-1 gB_eGC, allowing a direct comparison (35).

The expression of Cre as an independent protein by the new virus was confirmed by Western blotting (Fig. 3B), and this virus showed similar growth kinetics to the parent both *in vitro* and *in vivo* (Fig. 3C and D). To investigate the efficiency of neuronal marking when a T2A is used, groups of ROSA26 mice were infected with HSV-1 gB_eGTC_UL3/4 or HSV-1 gB_eGC. DRGs were stained to count the number of β-gal-expressing neurons at days 30, 100, and 200 after infection. We found that neither the number of β-gal^+^ cells nor the number of DRG with any β-gal^+^ cells were significantly different between the viruses at any day (Fig. 3E and F). These results show that T2A sequences can be used in recombinant HSV and independently verifies the detection of protein production from the gB promoter in latency from U_L_3/U_L_4.

### Protein expression from native promoters in latency

**Fig 4:**
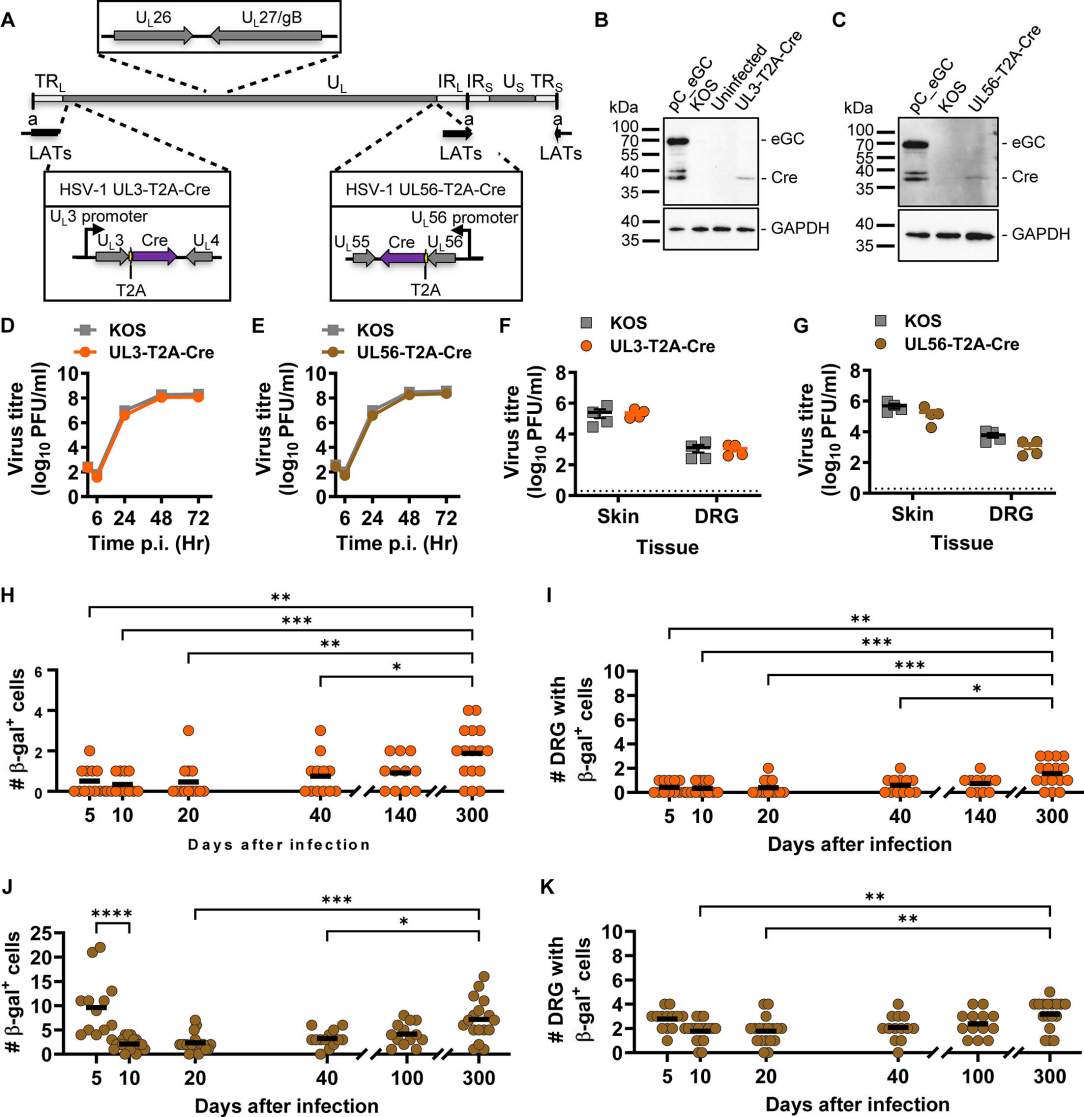
Promoter activity in latency is a feature of genomic location. (**A**) Schematic representation of the HSV-1 genome showing modifications made to generate UL3-T2A-Cre and UL56-T2A-Cre. (**B, C**) Detection of Cre and GAPDH by Western blotting; B and C share controls with Fig. 2B and Fig. 1C, respectively. (**D-G**) Replication of recombinants was assessed in comparison with parent KOS *in vitro* (**D, E**) and *in vivo* (**F, G**); E, F, and G share KOS controls with Fig. 1B, Fig. 3D and Fig. 1F, respectively. Symbols and markers are same as in previous figures. Statistical significance was determined using two-way ANOVA with Tukey’s *posttest* comparison (unmarked, not significant). (**H-K**) Groups of ROSA26R mice were infected with HSV-1 UL3-T2A-Cre (**H, I**) or UL56-T2A-Cre (**J, K**), their DRGs collected, and stained for assessing β-gal expression. The number of β-gal^+^ cells per mouse (**H, J**) and the number of DRG with at least one β-gal^+^ cell (**I, K**) are shown. Data are pooled from three independent experiments for each virus. Differences in means were compared using one-way ANOVA with Tukey’s *posttest* to calculate pairwise comparisons (*, *P* < 0.05; **, *P* < 0.01; ***, *P* < 0.001; ****, *P* < 0.0001).

Finally, we investigated the activity of two native promoters toward the ends of U_L_ where the expression of Cre from ectopic cassettes was able to be detected during latency. We chose U_L_3 and U_L_56, replacing their stop codons with a T2A, followed by Cre to make UL3-T2A-Cre and UL56-T2A-Cre, respectively (Fig. 4A). Both viruses expressed Cre as an independent protein (Fig. 4B and C) and had growth similar to the parent virus *in vitro* and *in vivo* (Fig. 4D through G). Finally, we used these viruses to examine the activity of native promoters in the Cre marking system. When Cre was driven from the native U_L_3 promoter, we found that β-gal^+^ neurons were either non-existent or very rare, limited to one or two neurons per mouse, and to single DRG on days 5 and 10 (Fig. 4H). This low level of marking was seen on days 20, 40, and 140, but there was a trend toward a higher mean and a greater fraction of mice having any marking with each later in time (Fig. 4H and I). At 300 days after infection, the average number of β-gal^+^ neurons and the number of DRG with marked neurons were significantly increased compared with all time points up to day 40 (Fig. 4H and I). Marking of neurons in ROSA26R mice by HSV-1 UL56-T2A-Cre was similar to that seen with UL3-T2A-Cre, except that an early peak was seen on day 5, before the number fell to very low levels on day 10. Thereafter, there was a trend of increased means that became statistically significant compared with days 10, 20, and 40 at day 300 (Fig. 4J). Similarly, the number of DRGs with at least one β-gal^+^ cell increased on day 300 compared with days 10 and 20 (Fig. 4K). These data show that the native U_L_3 and U_L_56 promoters can drive detectable Cre expression during HSV-1 latency.

## DISCUSSION

**Fig 5:**
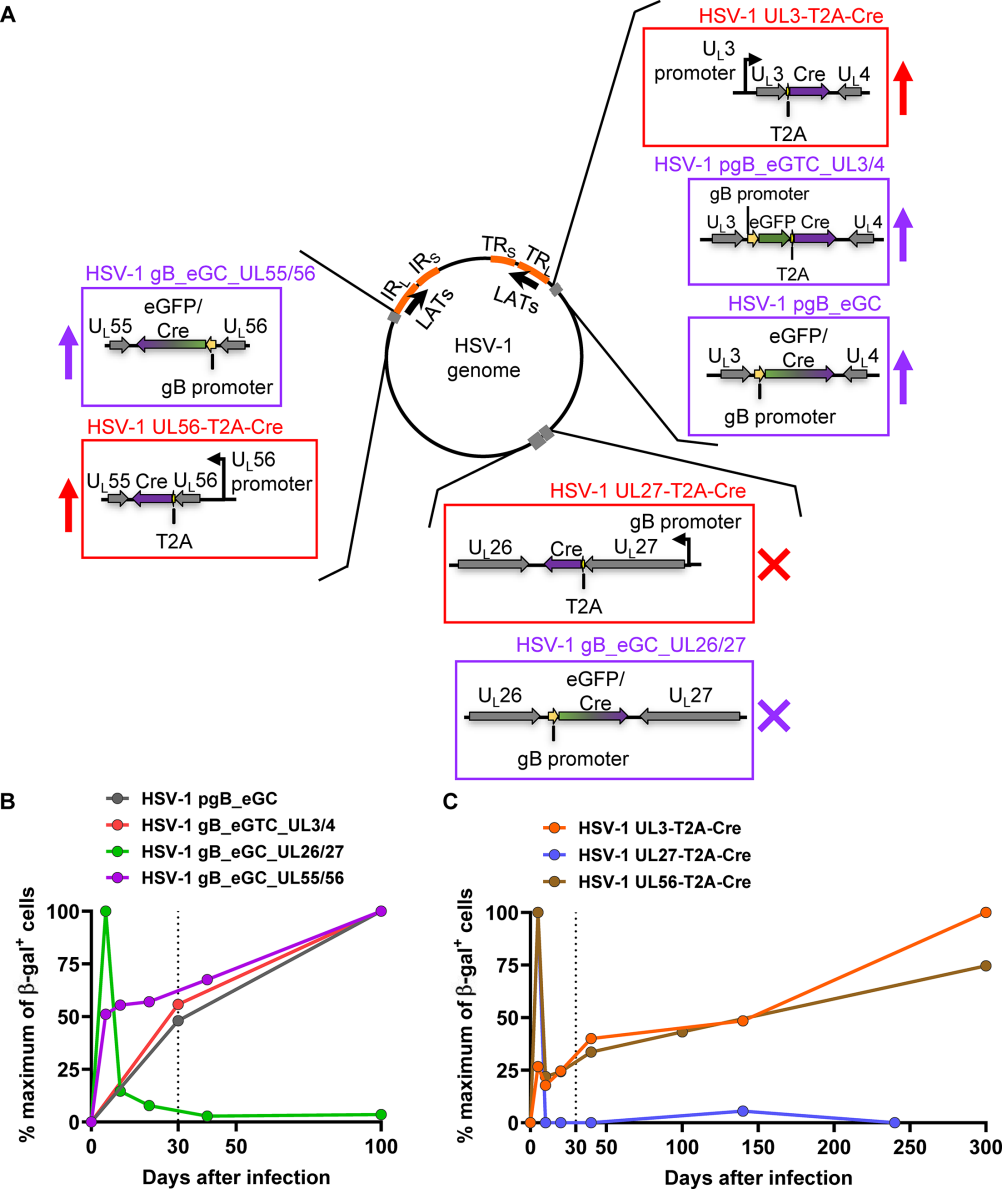
Summary of experiments where promoter activity was assessed in latency. (**A**) Schematic depicting the episomal form of the HSV-1 genome. The terminal and internal repeats and the LAT locus are marked (to scale). The recombinants used to study ectopic (purple box) and native (red box) promoters are shown. An upward arrow next to the box indicates that the promoter was active in latency, whereas a cross indicates no activity in latency. (**B, C**) The average of β-gal + numbers in DRGs of mice per time point is shown as the percentage of the maximum value (at the peak time point) from the viruses used to study ectopically placed gB promoter in (**B**) and native promoters in (**C**).

In this study, we used a Cre/ROSA26R mouse model to investigate the influence of location of a lytic promoter in the HSV-1 genome on its activity during latency, as well as the ability of natural lytic gene promoters to drive protein expression in latency. To achieve this, the gB promoter was studied in ectopic constructs from three locations: U_L_3/U_L_4, U_L_26/U_L_27, and U_L_55/U_L_56. In addition, the native promoters of gB, U_L_3, and U_L_56 were analyzed for their activity by using a T2A sequence to separate the HSV protein from Cre (45, 46). The results of neuronal marking by all the recombinant viruses used here have been summarized in Fig. 5, showing each as the percent of the maximum number of neurons marked to show the differences in kinetics clearly (Fig. 5). These show clearly that both for non-native and for native promoters, activity in latency was detectable from U_L_3/U_L_4 and U_L_55/U_L_56 at the ends of U_L_ but not from U_L_26/U_L_27, which lies in the middle of this segment.

Prior to this study, only two promoters have been studied using ROSA26R/Cre marking models when placed in two different locations. First, there was no accumulation of β-gal + cells in latency when Cre was driven by the ICP0 promoter, regardless of whether it was placed in the U_L_3/U_L_4 or within the U_S_5 gene (33, 35). This finding suggested that while a set of other promoters was active from U_L_3/U_L_4, placement in this location alone was not adequate to guarantee activity during latency (35). By contrast, ICP0 production from its natural location in latency has been inferred by a requirement for this protein for normal deposition of chromatin and expression of transcripts as read out at day 28 after infection (24). It remains to be determined if there is an ongoing requirement for this expression throughout latency. Second, while the promoter for ICP47 was active from U_L_3/U_L_4 during latency, this activity was not detectable from either the U_L_26/U_L_27 region or U_S_12, its native location (37). Taken together with our new data shown here, it would seem that a location near the LAT transcription region is necessary, if not sufficient to endow a lytic promoter with enough activity during latency that it can be detected in the ROSA26R/Cre model.

Latent genomes are organized into condensed chromatin structure, which is partly mediated by CTCF insulators that recognize and bind a conserved CCCTC motif in DNA (47, 48). CTCF proteins can self-dimerize, enabling them to bring spatially separated chromosomal regions into close proximity to form loops, known as topologically associated domains, further demarcating transcriptionally active (euchromatin) and inactive (heterochromatin) regions (49). During latency, the LAT region is notably characterized by the presence of transcriptionally active euchromatic marks (50–53), whereas neighboring lytic genes are associated with repressive heterochromatic marks (53–55). CTCF plays an essential role in establishing these chromatin states, and several CTCF-binding sites have been identified within the HSV genome (47, 48), particularly clustered around the LAT promoter/enhancer and IE genes (Fig. S5). Of particular importance are the CTCF-binding sites CTRL1 and CTRL2, which surround the LAT promoter (LAP1)/enhancer (LAP2) (Fig. S5). These sites were shown to be highly enriched in CTCF during latency, relative to all other sites (56, 57). Moreover, application of reactivation stimuli to mouse latently infected ganglia did not completely displace CTCF from these sites (56, 57), and upon depletion of CTCF from latently infected neurons of rabbits, increased viral reactivation was observed (58). These findings suggest that CTRL1 and CTRL2 sites strongly contribute to maintainance of latency by acting as insulators with LAT enhancer-blocking and silencing functions (59, 60). Consistent with this, loss of CTRL2 resulted in decrease of heterochromatin marks on adjacent promoters and an increase in the expression of lytic IE genes, including ICP0, which is transcribed antisense from within the LAT transcript, as well as ICP4 and ICP27 (U_L_54), which are adjacent to the LAT-expressing region (59, 61). These results indicate that CTRL2 has a strong LAT enhancer-blocking ability that extends as far as ICP27, which lies distal to U_L_55/U_L_56 tested here.

Irrespective of this enhancer-blocking ability of CTRL2 and generally repressive chromatin marks, our data suggest that leaky expression occurs, and this is most prevalent near the LAT enhancer. In line with this, the ICP47 promoter in its native location within R_S_, some 12.6 kb away from the LAT enhancer, was unable to drive the protein expression during latency (37). By contrast, a set of ICP47 promoter variants were all active in latency when driving *eGFP/Cre* from U_L_3/U_L_4 (37). Here, we extend this to the gB promoter that we find to be active at the ends, but not from the middle of U_L_. Further to this, the average number of marked neurons at day 100 was lower for gB_eGC compared with gB_eGC_UL55/56 (29 and 70, respectively). This order of marking for the gB promoter from these two locations corresponds to their distance from the LAT enhancer: The gB promoter in U_L_55/U_L_56 is 2.6 kb from the start of LAT transcription, whereas it is 4.1 kb for viruses that use U_L_3/U_L_4 (Table 1). Arguing against a simple correlation between distance from LAT and permissiveness for expression during latency is that the fold increase in β-gal^+^ cells from the start of latency to day 100 was higher for gB_eGC in U_L_3/U_L_4 than the U_L_55/U_L_56 region. This can be seen as a steeper slope for the gray compared to the purple line in Fig. 5B. Some caution is required here because these comments rely on comparisons across experiments, but we note that the slope for gB_eGC is similar here as in a previous publication (35) and also for the independently made gB_eGTC_UL3/4, suggesting the observations are likely to be robust. Finally, the association of repressive proteins, such as CTCF, is dynamic to allow for acute infection, latency, and reactivation (52, 57, 60, 62). Perhaps this instability is retained during latency in a subset of neurons, reflecting the relatively low level of marking seen for most promoters, similar to the restricted expression of LATs. It would be of interest to know if LAT and leaky lytic gene expression were correlated.

Future experiments could be carried out to delete the LAT enhancer and insulator elements, individually and together, to understand the impact of these on the activity of neighboring lytic genes in latency. It would also be of interest to know how far from the LAT enhancer detectable gene expression occurs and if this increased propensity for expression extends to ICP27, an essential immediate early gene, or to VP16 (U_L_48) that has been linked in some models to reactivation (63).

We chose to study the gB promoter in part because activated, gB-specific T cells are found in latently infected DRGs, which strongly suggests that the gB antigen is expressed during latency (64, 65). However, we were not able to detect Cre expression from this promoter, either when linked to the native gB with a T2A or from an additional copy of this promoter from the same part of the genome. Moreover, the average number of marked neurons that demonstrate survival of neurons after expression of the gB promoter at any time after infection was very low. The apparent inconsistency between the immunological studies of gB expression and ours could be attributed to the comparatively low sensitivity of our reporter system compared to the ability of T cells to detect extremely low levels of antigens. Where T cells may be able to detect as few as a handful of antigenic peptides, significantly higher levels of Cre are likely to be required to ensure migration to the nucleus, access to the target site, and recombination of the *loxP* sites (66–68).

Evidence for immunological detection of lytic antigens goes beyond gB and the phenotype of resident T cells to include persistent cytokine expression by infiltrating immune cells (69). Notably, the continual cytokine expression is present in the ganglia even after infection with a thymidine kinase-deficient virus that is unable to replicate and reactivate (69), suggesting that full engagement of the lytic cascade is not required for the activation of immune cells. Our observation that native late gene promoters, including a true-late gene (U_L_3), can drive protein expression in latency aligns with the idea that lytic gene expression during this phase of infection is de-coupled from the ordered cascade of productive infection. Whether this activity is a component of the animation phase of reactivation, which was never rendered complete (70–73), or a part of the latency program of gene expression involved in the maintenance of a latent state (36), remains to be elucidated.

Finally, we conclude that detection of native lytic gene promoter expression during latency using ROSA26R/Cre models can be interpreted simply as showing that these promoters can be active in latent infection. Our data also suggest that the activity is highest from promoters close to the LAT enhancer and that areas of the genome at the ends of U_L_ are not entirely insulated from the de-repression of the LAT region during latency. However, the failure to detect promoter activity in latency from across the genome requires more caution in interpretation and is better considered to be falling below the limit of sensitivity of the model,than to be entirely absent.

## MATERIALS AND METHODS

### Cell lines and viruses

Vero cells were obtained from American Type Culture Collection (ATCC, CCL-81). Cre reporter assays were performed in Vero SUA cells [gift from Prof Stacey Efstathiou (40)]. Both cell lines were cultured in minimum essential medium (MEM; ThermoFisher Scientific) supplemented with 2% or 10% heat-inactivated fetal bovine serum (FBS; Sigma-Aldrich), 4 mM L-glutamine (ThermoFisher Scientific), 5 mM HEPES buffer (ThermoFisher Scientific), and 55 µM β-mercaptoethanol (ThermoFisher Scientific). The transfections were carried out in 293A cells (ATCC, CCL-81) using Lipofectamine 2000 (ThermoFisher Scientific). 293A cells were cultured in Dulbecco’s modified Eagle medium (DMEM; ThermoFisher Scientific) supplemented with 10% heat-inactivated FBS (Sigma-Aldrich) and 2 mM L-glutamine (ThermoFisher Scientific).

HSV-1 KOS (38) was a gift from Dr. Francis R. Carbone (University of Melbourne, Australia), and all recombinant viruses used in this study were derived from the HSV-1 strain KOS. HSV-1 pC_eGC, pCmC, and gB_eGC have been described previously (39) (35). All viruses were titrated and grown on Vero cells as described elsewhere (39).

### Plasmid construction

The plasmid constructs used as the repair template for recombinant virus generation were generated using InFusion cloning (TaKaRa). The sequence references below are based on the HSV-1 KOS genome accession number JQ673480 (74). To construct pgB_eGC_UL26/27, promoter gB (55,985–56,282), eGFP/Cre, and bovine growth hormone (BGH) polyA termination sequence were amplified from pT gB_eGC (35), inserted into the *Spe*I site of pU26/7 (39). To construct pgB_eGC_UL55/56, flanking arms with U_L_55 (115,549–116,066) and U_L_56 (116,067–116,573) were amplified from HSV-1 KOS, and promoter gB, eGFP/Cre, and bovine growth hormone (BGH) polyA termination sequence were amplified from pT gB_eGC (35) and inserted into the *Bam*HI site of the pCR bluntII vector (Invitrogen, Life Technologies), and this plasmid was used to generate HSV-1 gB_eGC_UL55/56 (Fig. S1A).

To construct pgB_eGTC_UL3/4, the flanking U_L_3 and U_L_4 arms, gB promoter, and eGFP and Cre-BGH polyA sequences were amplified from pT gB_eGC (35). The T2A sequence was synthesized as two complementary dioxynucleotides (41). These fragments were cloned into the *Bam*HI site of the pCR bluntII vector (Invitrogen, Life Technologies), maintaining the original configuration as pT gB_eGC (35), except that T2A was inserted in frame between eGFP and Cre.

To construct pUL27-T2A-Cre, U_L_26 (52,391–53,024) and U_L_27 (53,025–53,391) containing flanking arms were amplified from HSV-1 KOS, T2A was synthesized as two complementary dioxynucleotides (41), and the Cre sequence was amplified from pT gB_eGC (35). These fragments were inserted into the *Bam*HI site of pCR bluntII vector (Invitrogen, Life Technologies) and this plasmid was used to generate HSV-1 UL27-T2A-Cre (Fig. S1B). To construct pUL3-T2A-Cre, T2A and Cre sequences were obtained as earlier, U_L_3 (11,184–11,610) and U_L_4 (11,611–12,049) containing fragments were amplified from HSV-1 KOS, and inserted into the *Bam*HI site of the pCR bluntII vector, and this plasmid was used to generate HSV-1 UL3-T2A-Cre (Fig. S1C). Plasmid UL56-T2A-Cre was constructed using U_L_55 (115,644–116,144) and U_L_56 (116,145–116,660) fragments amplified from HSV-1 KOS and the T2A-Cre fragment from pUL27-T2A-Cre, all cloned into the *Bam*HI site of the pCR bluntII vector, and this plasmid was used to generate HSV-1 UL56-T2A-Cre (Fig. S1D).

The pX330 plasmid has been described previously (75) and was purchased from Addgene (plasmid 42230). The sequences coding for appropriate guide RNA were synthesized as two complementary dioxynucleotides, annealed to generate double-stranded DNA fragments, and inserted into the *Bbs*I site of pX330. Oligonucleotides used to generate pX330-UL55-56 are CACCGCCAGGCGTGGTGTGAGTTTG and AAACCAAACTCACACCACGCCTGGC, those for pXUL26/27 are CACCTTTGTCACGGGAAAGGAAAG and AAACCTTTCCTTTCCCGTGACAAA, those for pX330-UL27 are CACCGCCGACGAGGACGACCTGTGA and AAACTCACAGGTCGTCCTCGTCGGC, and those for pX330-UL56 are CACCGACAGGGGCGCTTACCGCCAC and AAACGTGGCGGTAAGCGCCCCTGTC.

### Recombinant virus generation and *in vitro* growth analysis

All the recombinant viruses were constructed using a transfection/infection method described previously (39, 76). To construct HSV-1 gB_eGC_UL26/27 or gB_eGC_UL55/56, linearized pgB_eGC_UL26/27 was cotransfected with pXUL26/27 or pgB_eGC_UL55/56 was cotransfected with pXUL26/27, respectively, into Vero cells, followed by infection with HSV-1 KOS. HSV-1 gB_eGTC_UL3/4 or UL3-T2A-Cre was constructed after cotransfecting pgB_eGTC_UL3/4 or UL3-T2A-Cre, respectively, with pX330-mC, followed by infection with HSV-1 pCmC (39). To construct HSV-1 UL27-T2A-Cre or UL56-T2A-Cre, Vero cells were cotransfected with pUL27-T2A-Cre and pX330-UL27 or pUL56-T2A-Cre and pX330-UL56, respectively, following infection with HSV-1 KOS. All the viruses used in this study have been listed in Table 1.

The screening of the desired recombinant was carried out based on fluorescence where possible or PCR. Each virus was purified further via three rounds of plaque purification, and the absence of the parent virus in the final round was confirmed by PCR. The introduced modification in the recombinant was verified by PCR, Sanger sequencing, and restriction fragment length polymorphism (Fig. S2). The expression of Cre was verified by Western blotting, and the function was validated by Vero SUA reporter assays (Fig. S3).

The growth kinetics of newly generated viruses was checked in comparison with the parent virus KOS in Vero cells, as described previously (35). In some experiments, two viruses that are presented in different figures were tested for growth *in vitro* with a single control (KOS), so the control data are shown twice, once with each test virus. The relevent panels are Fig. 1D and 3C; Fig. 1E and 4E.

### Mice and infections

At least 8-week-old, female, specific-pathogen-free C57BL/6 or B6.129S4-Gt(ROSA)26Sortm1So/J (ROSA26R) (32) were used for experiments. The mice were housed and bred at APF. ROSA26R mice were a gift from Dr. Francis R. Carbone (University of Melbourne, Australia). Mice were anesthetized by intraperitoneal injection with Avertin (2,2,2-tribromoethanol in 2-methyl-butanol) or ketamine/xylazine mix before infection with 1 × 10^8^ PFU/mL of virus on the shaved flank. The procedure was followed as described previously, except that the needle was charged for 20 seconds in the virus suspension and tattooed for a period of 20 s (39). The virus dose and infection route were the same for all the experiments.

### Virus titration from skin and DRGs

Mice were euthanized with an increasing concentration of CO_2_, the infected area of the skin (2.5 cm vertically ×0.8 cm horizontally) and the DRG (T5-L1) on the ipsilateral side were excised, and collected in 500 µL MEM (without serum) 5 days after infection. The tissue samples were snap-frozen, 5-mm-diameter stainless steel bead (Qiagen) was added to all the tubes, and the tissue was homogenized in TissueLyser II (Qiagen) at an oscillation frequency of 30 Hz for 90 seconds twice. Homogenates were subjected to three freeze/thaw cycles, and the amount of infectious virus was quantified by a standard plaque assay on Vero cells. In some experiments, two viruses that are presented in different figures were tested for growth *in vivo* with a single control (KOS), so the control data are shown twice, once with each test virus. The relevent panels are Fig. 1F and 4G; Fig. 3D and 4F.

### Detection of β-gal expression

To check the expression of β-gal *in vitro*, confluent Vero SUA cells were left untreated or infected with the appropriate virus at a multiplicity of infection (MOI) of 0.05 PFU/cell and incubated at 37°C with 5% CO_2_ for 1 hour. The unabsorbed virus was removed, replaced with fresh medium (MEM with 2% serum), and cells were further incubated for 36 hours. Following incubation, the cells were washed with PBS (Sigma-Aldrich), fixed with 2% paraformaldehyde (Electron Microscopy Sciences)/ 0.5% glutaraldehyde (Sigma-Aldrich) (in PBS) for 4 hours at 4°C, washed with PBS again, and incubated in the permeabilization solution (2 mM magnesium chloride (Ajax FineChem), 0.01% (wt/vol) sodium deoxycholate (Sigma-Aldrich), 0.02% (vol/vol) IGEPAL CA-630 (Sigma-Aldrich), 5 mM potassium ferrocyanide (Sigma-Aldrich), and 5 mM potassium ferricyanide (Sigma-Aldrich) in PBS) containing 1 mg/mL X-gal (Sigma-Aldrich; prepared fresh as a 40 mg/mL stock in *N,N*-dimethylformamide (Sigma-Aldrich)) overnight at 4°C. Cells were washed with PBS, overlayed with 50% glycerol (Sigma-Aldrich; in PBS), and then visualized and imaged using an Olympus CKX41 microscope fitted with an Olympus DP22 digital camera.

To enumerate β-gal-expressing cells *in vivo*, mice were euthanized with an increasing concentration of CO_2_, and the DRGs (T5-L1) on the ipsilateral side were collected individually in a fixative as above. The DRGs were incubated on ice for 1 hour and then washed twice with PBS before adding the permeabilization buffer as before. Following further incubation at 4°C for 30 minutes, the solution was replaced with permeabilization buffer containing 1 mg/mL X-gal (prepared as above), and DRGs were incubated at 4°C for 12–16 hours. DRGs were washed with PBS again and incubated in 50% glycerol (in PBS) overnight. The DRGs were visualized and imaged using an Olympus CKX41 microscope equipped with an Olympus DP22 digital camera. The β-gal^+^ cells were either counted manually or with the aid of ImageJ software (77).

### Restriction fragment length polymorphism

For extracting viral DNA, 80% confluent Vero cells were infected with appropriate virus in a 175-cm^2^ area flask at an MOI of 0.05 PFU/cell for 1 hour at 37°C with 5% CO_2_. The fresh medium was added, and cells were further incubated for 48 hours. The cells and supernatant were centrifuged to remove the cell debris. The supernatant was further centrifuged at 17,684 × *g* for 90 mins at 4°C, and the pellet obtained was resuspended in TE-SDS (10 mM Trizma base (pH 8.0) (Sigma-Aldrich), 1 mM EDTA (ThermoFisher Scientific), and 0.5% (wt/vol) SDS (Sigma-Aldrich) in water). DNA was extracted from the lysate using a standard phenol and chloroform extraction method (78), digested with an appropriate restriction enzyme, and electrophoresed on an agarose gel (Fig. S2).

### Western blotting

Confluent Vero cells were infected with appropriate virus at an MOI of 10 PFU/cell and incubated for 20 hours at 37°C with 5% CO_2_. Cells were washed once with PBS and resuspended in RIPA buffer (200 mM Trizma base (Sigma-Aldrich), 150 mM sodium chloride (Bacto), 1% Triton X-100 (Sigma-Aldrich), 0.5% sodium deoxycholate (Sigma-Aldrich), 0.1% SDS (Sigma-Aldrich), and 1 protease inhibitor tablet (Merck) per 10 mL of water, pH adjusted to 7.4). The amount of protein in the lysate was quantified using the Pierce BCA Protein Assay Kit (ThermoFisher Scientific) according to the manufacturer’s instructions. About 20 µg of protein was separated by sodium dodecyl sulfate-polyacrylamide gel electrophoresis (SDS-PAGE) and transferred onto the Immobilon-P PVDF membrane (Merck), followed by blocking in milk and further overnight probing at 4°C with a primary antibody specific for Cre (1:500; Cell Signaling Technology, mAb #12830) or GAPDH (1:5000; Cell Signaling Technology, mAb #5174). A goat anti-rabbit horseradish peroxidase (HRP)-conjugated secondary antibody (1:10,000; Jackson ImmunoResearch Laboratories Inc., AB_2313567) was applied to the membrane for 1 hour at room temperature. The membranes were imaged using a chemiluminescence detection system (ChemiDoc MP, Bio-Rad) after treating with an HRP substrate (Clarity Western ECL substrate, Bio-Rad) according to the manufacturer’s instructions. The six new viruses were tested for Cre expression across three blots, each having two viruses and a shared set of controls. In each case, the viruses tested together are presented in different figures, so the controls are shown twice, once with each test virus. The relevent panels are Fig. 1B and 3B; Fig. 1C and 4C; Fig. 2B and 4B.

### Statistical analysis

Statistical comparisons of means were done using a one-way or two-way analysis of variance (ANOVA) with an appropriate *posthoc* test in GraphPad Prism (Version 10.0.1). A *P* value less than 0.05 was considered significant.

## Data Availability

All data in this study are presented here as main and supplemental figures or tables.

## SUPPLEMENTAL MATERIAL

Supplemental figures jvi.01258-24-s0001.pdf Figures S1 to S5.

ASM does not own the copyrights to Supplemental Material that may be linked to, or accessed through, an article. The authors have granted ASM a non-exclusive, world-wide license to publish the Supplemental Material files. Please contact the corresponding author directly for reuse.
