# Supplementary material for: Lytic promoter activity during herpes simplex virus latency is dependent on genome location
Source: J Virol. 2024 Oct 21;98(11):e01258-24. doi: 10.1128/jvi.01258-24 (PMC11575402; doi:10.1128/jvi.01258-24)
Supplement: Supplemental figures — Figures S1 to S5. [file jvi.01258-24-s0001.pdf]

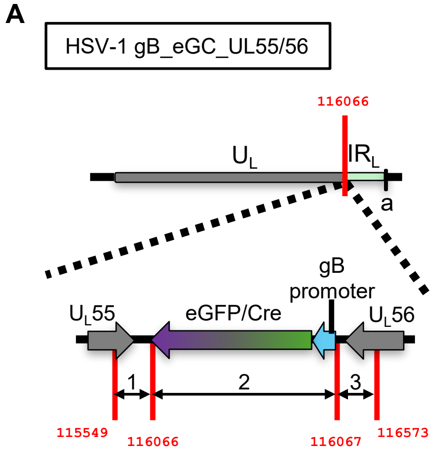

Fragment	Insert	Source
1	U_L55 flank	HSV-1 KOS
2	gB-eGC-polyA	pT gB_eGC (Russell and Tscharke 2016)
3	U_L56 flank	HSV-1 KOS

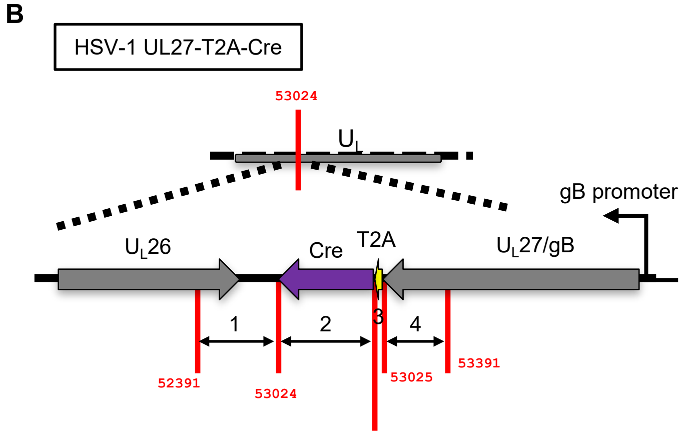

Fragment	Insert	Source
1	U_L26 flank	HSV-1 KOS
2	Cre	pT gB_eGC (Russell and Tscharke 2016)
3	T2A	Liu et al. 2017, Figure 1
4	U_L27 flank	HSV-1 KOS

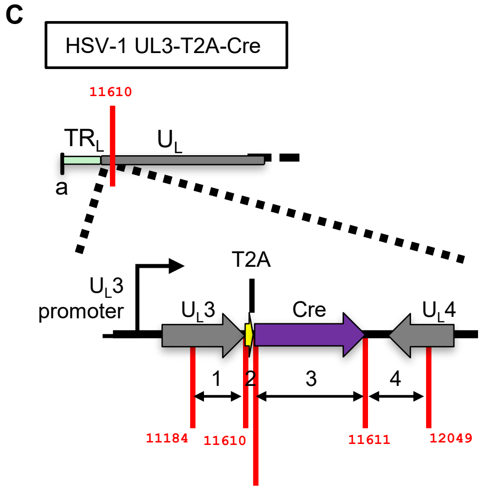

Fragment	Insert	Source
1	U_L3 flank	HSV-1 KOS
2	T2A	Liu et al. 2017, Figure 1
3	Cre	pT gB_eGC (Russell and Tscharke 2016)
4	U_L4 flank	HSV-1 KOS

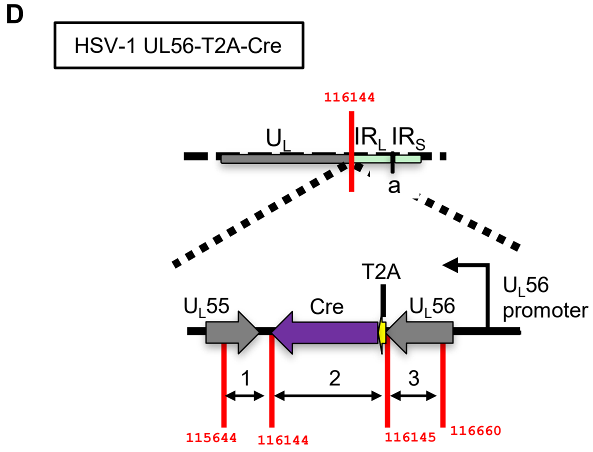

Fragment	Insert	Source
1	U_L55 flank	HSV-1 KOS
2	T2A-Cre	pUL27-T2A-Cre
3	U_L56 flank	HSV-1 KOS

Figure S1: Design of HSV-1 viruses that express Cre.
Schematic representation of the HSV-1 genome showing the insertion point where gB-eGC cassette was inserted to make gB_eGC_UL55/56 (A), and T2A-Cre cassette was inserted to make UL27-T2A-Cre (B), UL3-T2A-Cre (C) and UL56-T2A-Cre (D). The insertion site has been expanded to show the source of the fragments to make the repair plasmid used to make these viruses and the base pair position of insertion is indicated using HSV-1 KOS genome (JQ673480). T2A sequence was obtained from figure 1, Liu et al. 2017 (Liu, Ziqing, et al. "Systematic comparison of 2A peptides for cloning multi-genes in a polycistronic vector." *Scientific reports* 7.1 (2017): 2193.) and pT gB_eGC was obtained from Russell and Tscharke 2016 (Russell, Tiffany A., and David C. Tscharke. "Lytic promoters express protein during herpes simplex virus latency." *PLoS pathogens* 12.6 (2016): e1005729.)

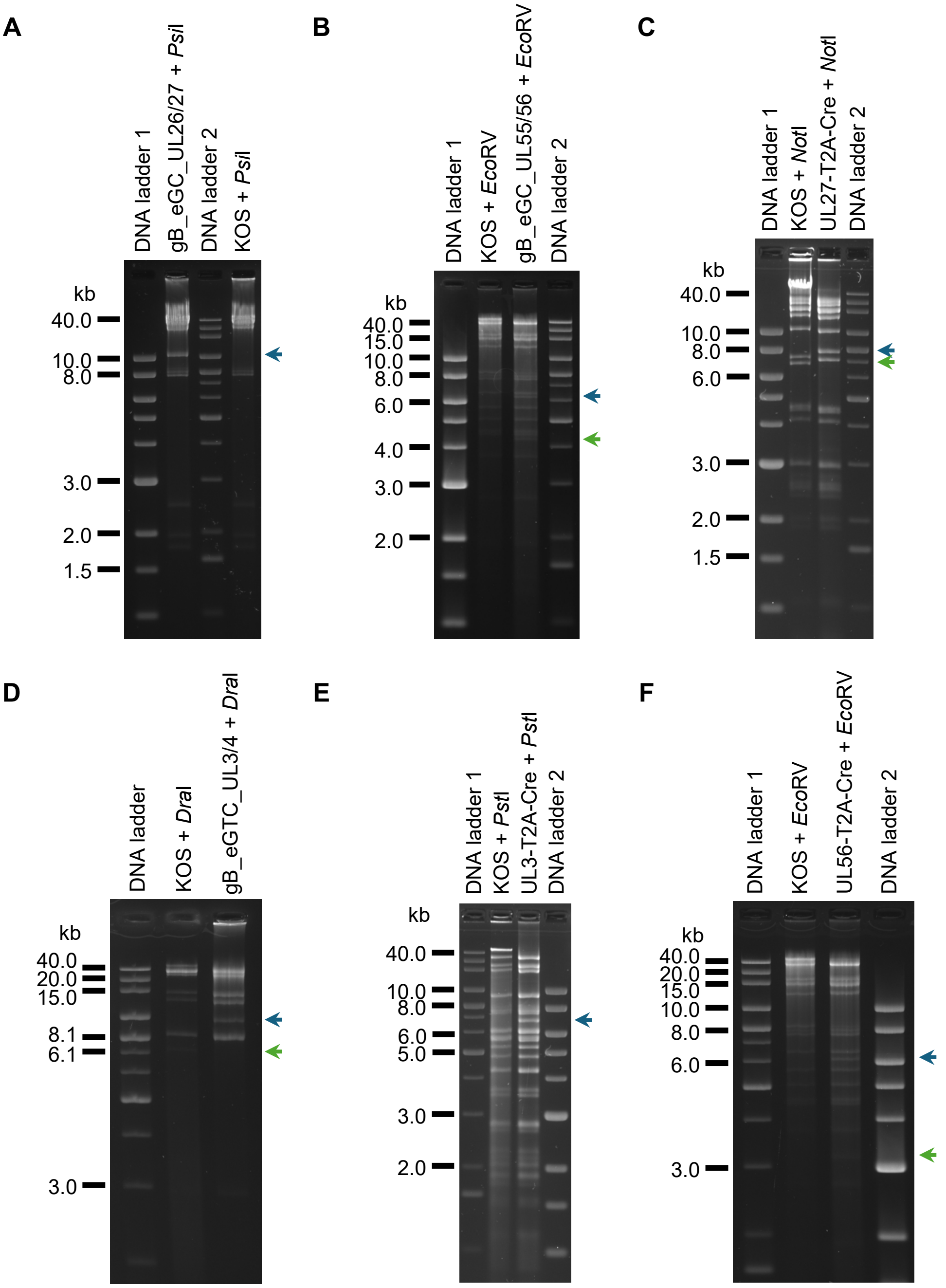

Figure S2: Verification of insertion location in HSV-1 recombinants using a whole genome digest.

The insertion location was verified by digesting the genome of HSV-1 recombinants gB_eGC_UL26/27 (A), gB_eGC_UL55/56 (B), UL27-T2A-Cre (C), gB_eGTC_UL3/4 (D), UL3-T2A-Cre (E), UL56-T2A-Cre (F), and compared with parent KOS. The size of DNA fragments from ladders are labelled on the left and arrows on the right indicate fragments of difference between the parent and recombinant.

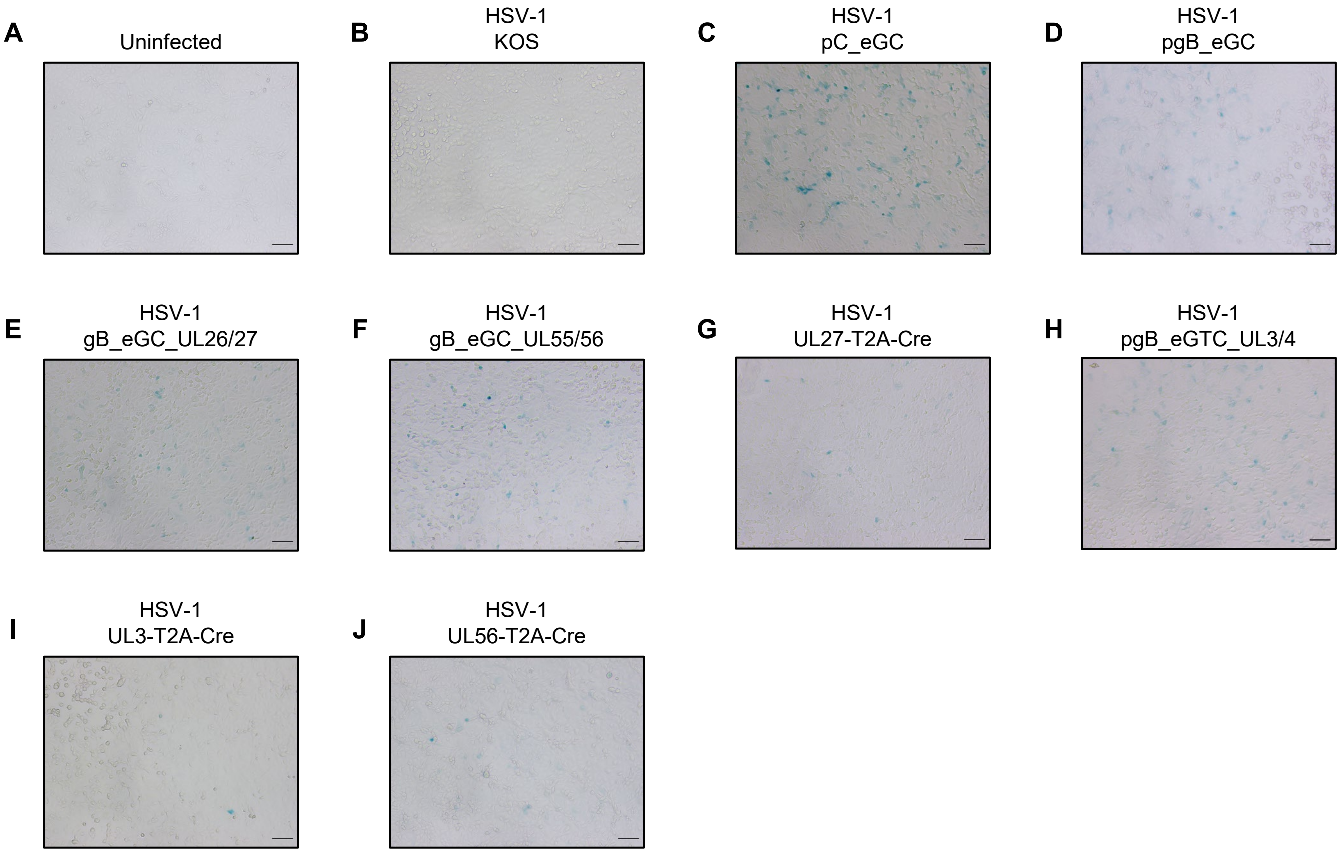

Figure S3: Functional assessment of Cre expressed by various recombinants.
Vero SUA cells were either left uninfected (A) or infected with HSV-1 KOS (B), pC_eGC (C), gB_eGC (D), gB_eGC_UL26/27 (E), gB_eGC_UL55/56 (F), UL27-T2A-Cre (G), gB_eGTC_UL3/4 (H), UL3-T2A-Cre (I) and UL56-T2A-Cre (J), at 0.05 PFU/cell for 1 hr and incubated further for 36 hrs. The cells were stained for β-gal activity before imaging (scale bar = 100 μm).

A **HSV-1 gB_eGC_UL26/27**

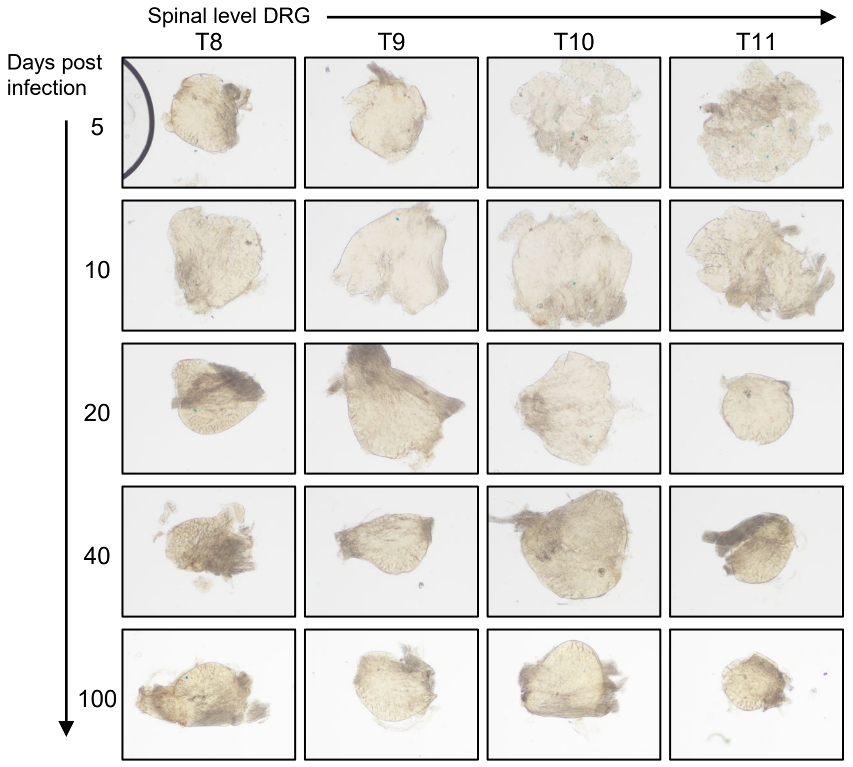

B **HSV-1 gB_eGC_UL55/56**

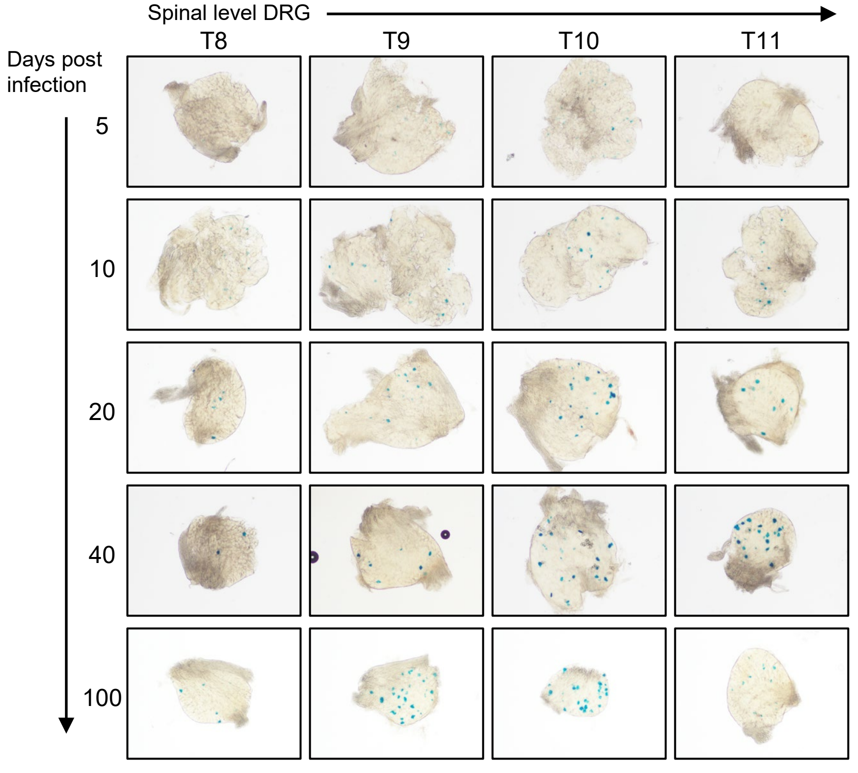

Figure S4: Determination of number of β-gal⁺ cells in the DRG of mice infected with recombinant viruses.

Groups of ROSA26R mice were infected with HSV-1 gB_eGC_UL26/27 or gB_eGC_UL55/56, and their DRG (T5-L1) were isolated and stained for β-gal activity. Representative photomicrographs of DRG (T8-T11) of a single mouse for each day are shown.

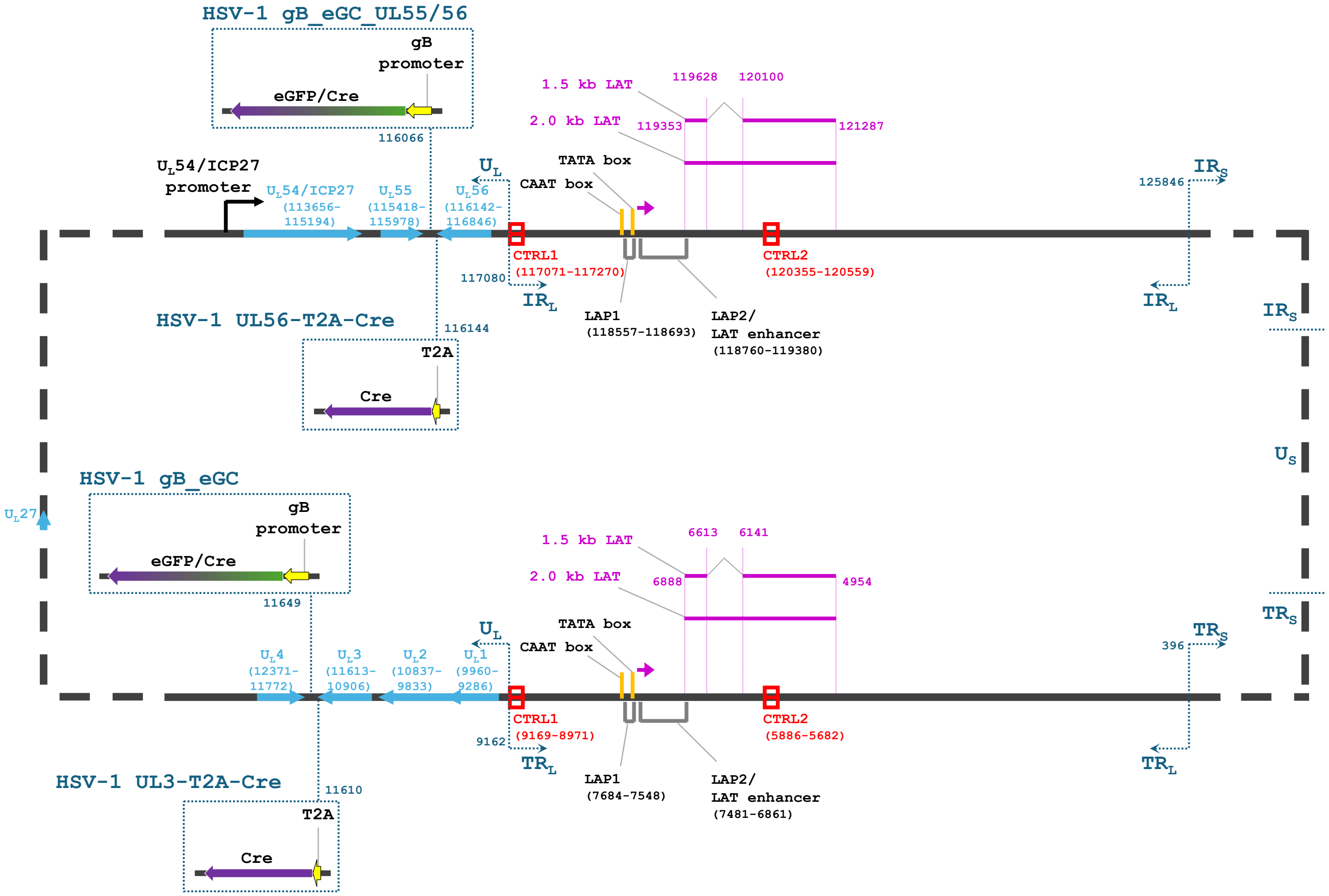

Figure S5: Structural map of HSV-1 LAT locus.

Schematic representation of the LAT locus and neighbouring genes in the HSV genome to allow alignment of U_L/IR_L (top) and U_L/TR_L (bottom) junctions of standard genomic isomer of HSV-1. The region shown as a hard line is drawn to scale and base pair positions (inclusive for each element) are from HSV-1 KOS genome (JQ673480). LAT promoter (LAP1), consisting of CAAT and TATA box, followed by LAT enhancer (LAP2) are indicated. Direction of LAT transcription and major LATs are shown in pink. Genomic position of CTCF binding sites CTRL1 and CTRL2 are shown in red. Other features present include genes adjacent to CTRL1 (in blue) and the insertion sites used to generate recombinant viruses in this study.
